# Odontogenic Keratocyst of the Anterior Mandible Mimicking Lateral Periodontal Cyst

**DOI:** 10.7759/cureus.94112

**Published:** 2025-10-08

**Authors:** Shilpa Mathew, Roshni Ramesh, S. Santhosh Kumar

**Affiliations:** 1 Periodontics, Government Dental College Thiruvananthapuram, Thiruvananthapuram, IND; 2 Periodontics, Government Dental College Alappuzha, Alappuzha, IND; 3 Periodontics, Government Dental College Kottayam, Kottayam, IND

**Keywords:** enucleation, histopathology, odontogenic keratocyst, periodontal cyst, platelet-rich fibrin

## Abstract

Odontogenic keratocyst (OKC) is thought to be an odontogenic cyst of developmental origin that can arise at any place in the jaws, but mostly occurs in the mandibular angle and ramus area. It is clinically indistinguishable from a lateral periodontal cyst. A proper diagnosis can only be made based on histopathological examination. A 47-year-old woman presented with a gingival swelling on the inner aspect of the anterior region of the lower jaw. The swelling was of one year's duration and asymptomatic. Intraoral periapical radiograph revealed a well-defined oval radiolucency in the interradicular area of the lower left mandibular incisors. Cone-beam computed tomography revealed destruction of the lingual cortical plate. A provisional diagnosis of lateral periodontal cyst was made. The lesion was enucleated, and the bone defect was filled with advanced platelet-rich fibrin. Histopathological examination confirmed a diagnosis of OKC. Patient follow-up after three years showed no recurrence. By reporting this rare location of OKC, the authors would like to emphasize that there is a vast variety of maxillofacial bone lesions that resemble each other clinically and radiographically, and hence, a histopathological examination should be done for a definitive diagnosis.

## Introduction

Odontogenic keratocyst (OKC) was first explained by Philipsen (1956) and subsequently established by Browne [1,2]. Initially, it was opined to be a benign but most likely to be an aggressive and recurrent odontogenic cyst [3,4]. OKC is also called a primordial cyst because the dental lamina (DL), which is hypothesized in its pathogenesis, is considered primordial epithelium [5].

An OKC that occurs between the roots of the premolars is known as a lateral OKC and may resemble a lateral periodontal cyst (LPC) or a lateral radicular cyst [6]. Later in 2005, the World Health Organization (WHO) classified OKC as keratocystic odontogenic tumors (KCOTs) due to the neoplastic nature and tumor-like characteristics of the lining epithelium [4]. However, in the latest WHO Classification of Head and Neck Tumors (2017), it was renamed as odontogenic keratocyst [7].

The characteristic features of OKCs are their potentially aggressive behavior, distinct histopathological findings, and high recurrence rate. It may also be seen associated with nevoid basal cell carcinoma or Gorlin syndrome [8]. OKC usually affects the mandible rather than the maxilla. In the mandible, the posterior areas, especially the angle, body, and ramus, are involved [9]. OKCs may present with symptoms of pain, swelling, and discharge, but can also be asymptomatic. A male predilection is reported, with a male-to-female ratio of 1.6:1.8. The typical age of occurrence of OKC is the second and third decades of life [8].

Radiographically, OKCs can present as unilocular or multilocular radiolucency with well-defined sclerotic borders. Unilocular OKCs that are located between the roots of teeth can mimic an LPC [10]. LPCs are nonkeratinized developmental cysts observed lateral to or adjacent to the root of a vital tooth [11]. LPCs are diagnosed based on clinical and histopathological findings. This article aims to report an atypical case of OKC located in the lingual aspect of the mandibular anterior region in a female patient in her late 40s, which clinically and radiographically mimicked an LPC. The surgical management of the lesion is also discussed. The rarity of this case is the unusual location of the lesion and its occurrence in an older patient. The clinical relevance lies in the aggressive behavior and high recurrence rate, mandating regular follow-up.

## Case presentation

A 47-year-old female patient reported to the department of periodontology with a swelling in relation to the inner aspect of the lower front gum region for one year. There was no history of pain, fever, or paraesthesia. She gave a history of spontaneous disappearance and reappearance of the swelling. The patient did not notice any increase in the size of the swelling. The patient has been under treatment for thyroid disease for the past few years. She did not have any other medical history.

**Figure 1:**
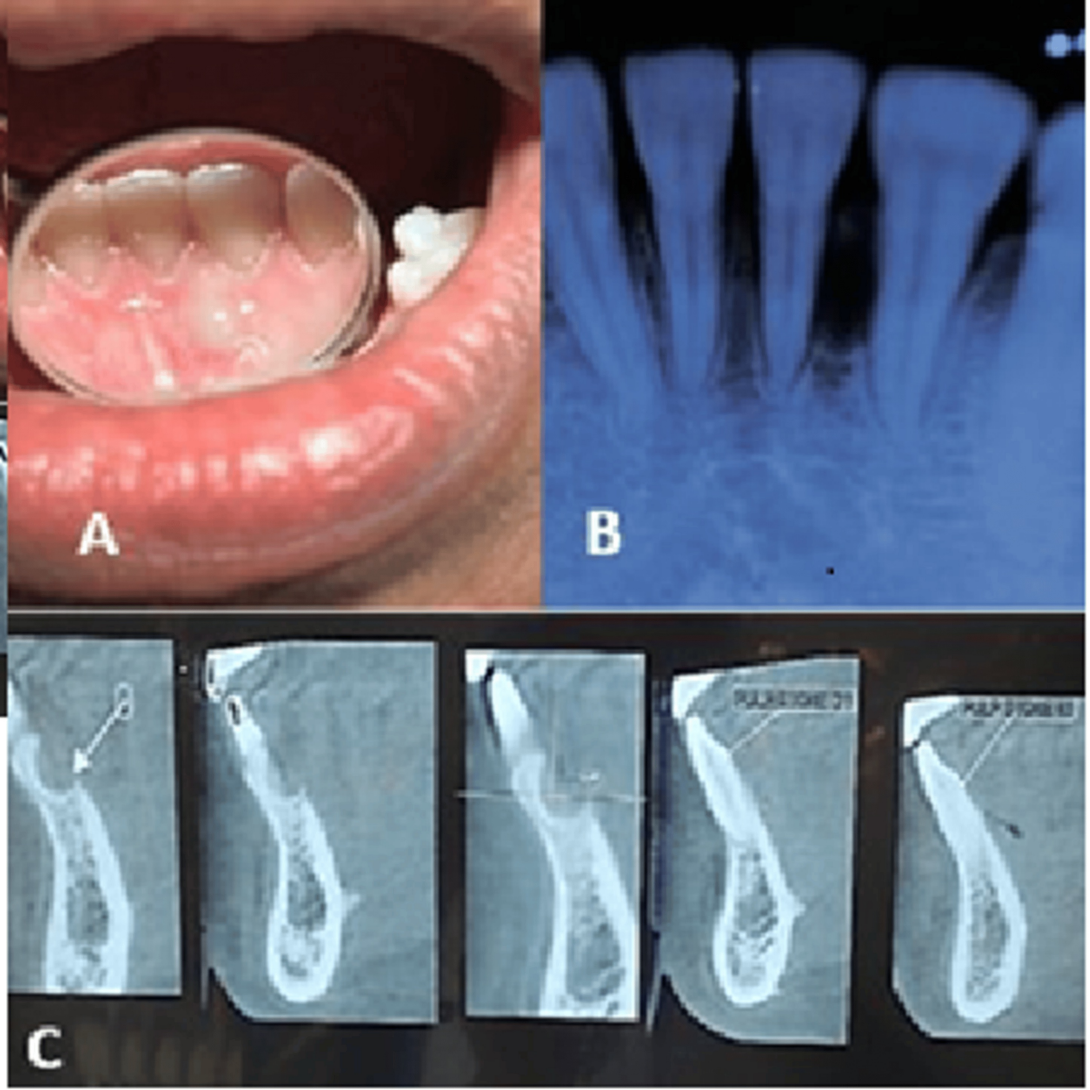
(A) Preoperative view of the swelling from the lingual aspect. (B) Preoperative intraoral periapical radiograph of the teeth 31 and 32 region. (C) Cone-beam computed tomography view showing thinning and perforation of the lingual cortical bone.

On clinical examination, an ovoid swelling of size 2 × 1 cm was seen between teeth # 31 and 32 on the lingual aspect (Figure 1A). The tissue over the swelling was pale pink with a slight bluish hue. The swelling had a non-inflammatory gingival appearance. The borders of the swelling were not well defined. On palpation, it was firm to hard in consistency. The swelling was non-tender, and the teeth were vital upon electric pulp testing. Both the teeth were non-mobile. Extraoral examination did not reveal any clinically discernible asymmetry, swelling, or lymphadenopathy. An intraoral periapical (IOPA) radiograph of the site revealed a well-defined unilocular interradicular radiolucency with a sclerotic border involving the mid root region between teeth # 31 and 32 (Figure 1B). The cone-beam computed tomography (CBCT) image showed thinning and perforation of the lingual cortical plate (Figure 1C).

Based on clinical and radiological findings, a provisional diagnosis of lateral periodontal cyst was made. Routine blood investigations were done, which showed normal values.

The lesion was suspected in the first place to be a lateral periodontal cyst, predicated on the presentation of the lesion clinically and radiographically. As in the case of a lateral periodontal cyst, the lesion was painless, non-inflammatory, and was found in close proximity to the roots of vital teeth. Other differential diagnoses included dentigerous cyst, OKC, ameloblastoma, traumatic cyst, giant cell granuloma, and odontogenic myxoma. Regardless of the elicited diagnosis, the treatment objective is conservative surgical management and histopathological examination. Therefore, a complete surgical enucleation of the lesion under local anesthesia was planned.

**Figure 2:**
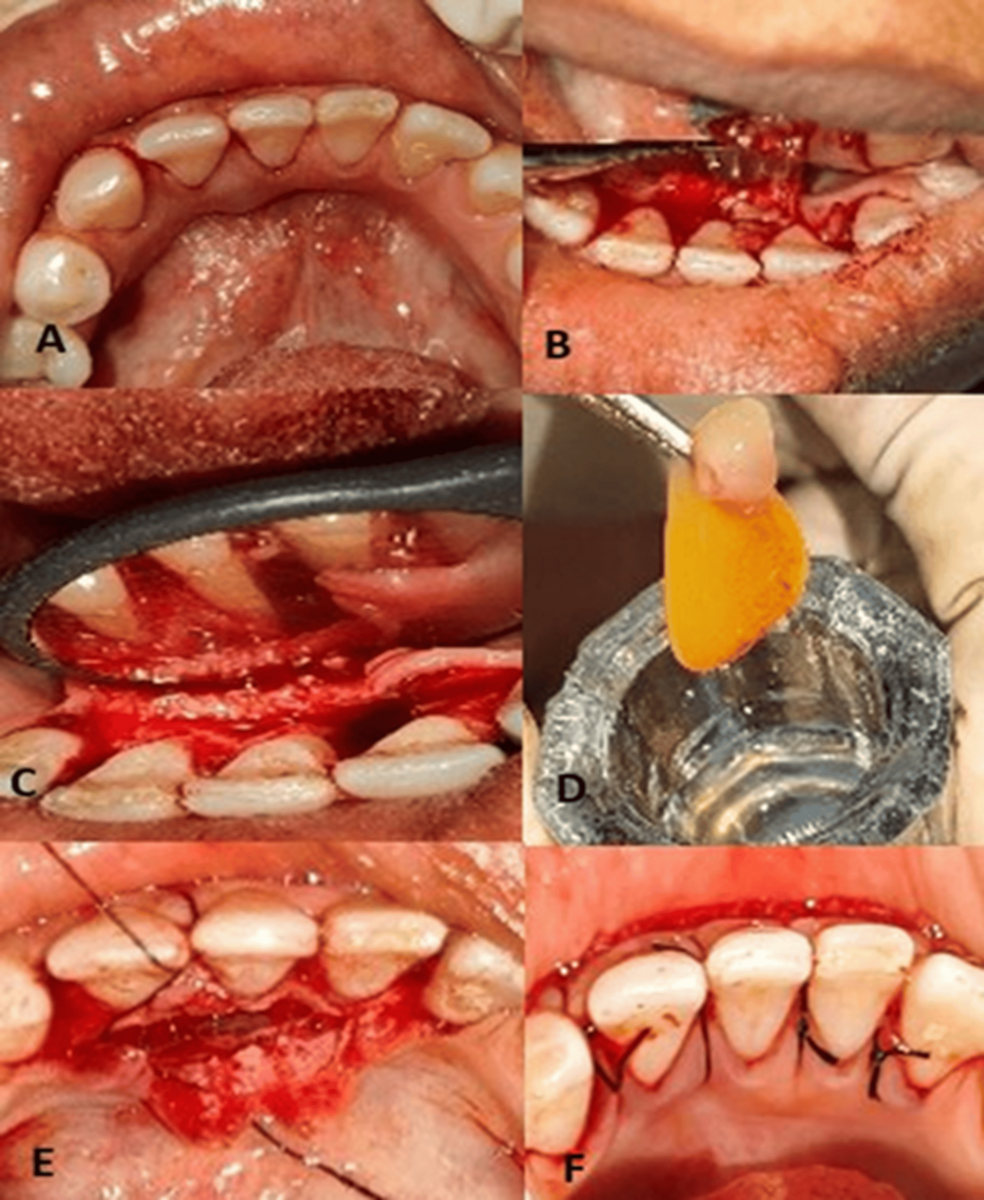
(A) Intra-crevicular incision was made. (B, C) Exposed full-thickness mucoperiosteal flap. (D) An advanced platelet-rich fibrin (A-PRF) gel was prepared. (E) A-PRF with Bio-Oss graft placed in the defect. (F) Sutures were placed.

Written informed consent was obtained from the patient for non-surgical and surgical treatment and for publication of the results. Initial scaling and root planing were done. After two weeks post scaling, conservative surgical removal of the lesion was done. With all aseptic precautions in the dental clinic, local anesthesia was administered to the patient. An initial intra-crevicular incision was made (Figure 2A). A mucoperiosteal flap was raised, and the lesion was completely enucleated along with cystic lining and contents (Figures 2B, 2C). Thorough irrigation of the cystic cavity was done using normal saline. Advanced platelet-rich fibrin (A-PRF) gel was prepared by drawing 10 ml of blood from the patient by a trained phlebotomist (Figure 2D). A-PRF gel, along with Bio-Oss bone graft, was placed into the cystic cavity (Figure 2E), and the mucoperiosteal flaps were sutured using 4-0 silk sutures (Figure 2F).

**Figure 3:**
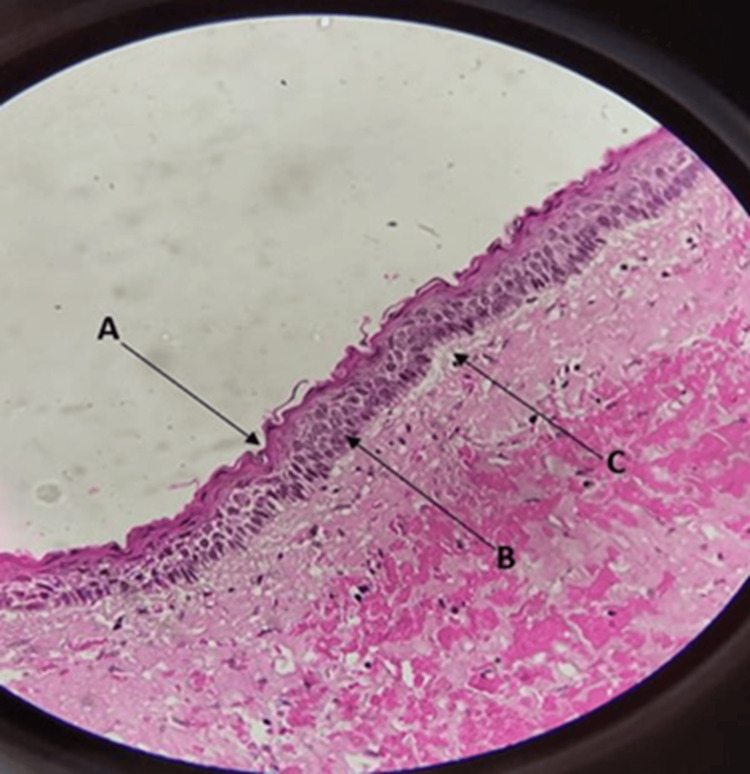
Histopathologic view of the lesion showing (A) uniform, corrugated, para-keratinized, stratified, squamous odontogenic epithelium overlying a fibrous stroma. (B) Hyperchromatic, palisading basal cells of the epithelium are conspicuous. (C) Epithelial separation from connective tissue is seen (H&E-stained magnification, 100x).

Histopathological examination using hematoxylin & eosin (H&E) stain revealed a five to six-layered corrugated, para-keratinized, stratified, squamous odontogenic epithelium overlying a connective tissue stroma of moderate to dense collagenicity and moderate cellularity (Figure 3A). The epithelial connective tissue interface was flat in some areas, and in other areas, the epithelium was seen separated from the underlying connective tissue stroma (Figure 3C). The basal cells of the epithelium showed a palisading pattern (Figure 3B). Vascularity of the stroma was moderate with formed, forming, and engorged blood vessels. Numerous extravasated RBCs were also noted within the cyst wall. Inflammatory response was minimal. The overall histopathological findings were suggestive of an OKC (Figure 3). Based on the clinical findings, radiographic appearance, and the histopathologic features, a final diagnosis of OKC was made.

**Figure 4:**
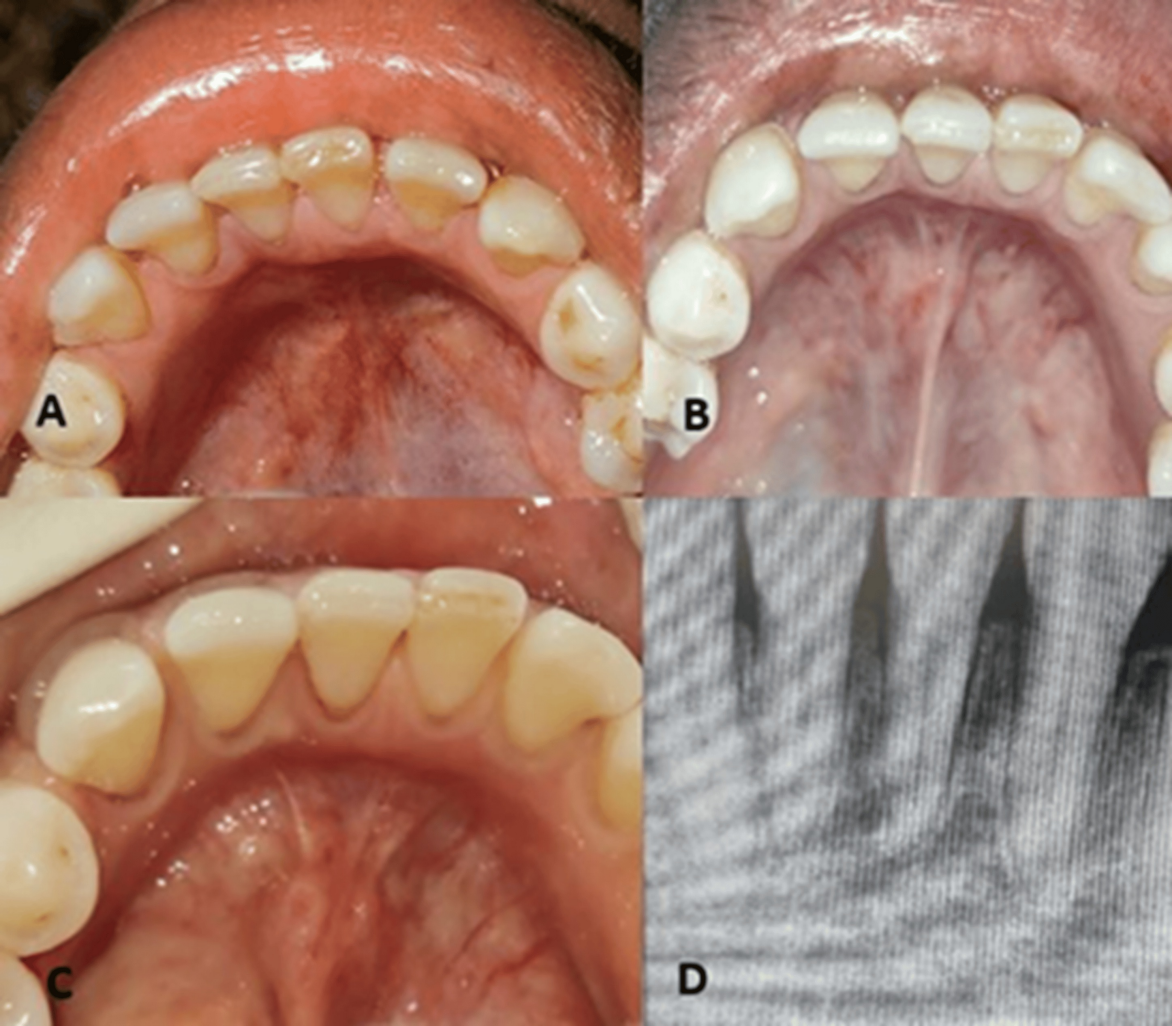
Review of the patient after (A) three months, (B) one year, and (C) three years. (D) Intraoral periapical radiograph after three years.

The patient was regularly followed up at one, three, six, 12, 24, and 36 months. A three-year clinical and radiographic follow-up showed complete recovery with no signs of recurrence (Figure 4).

## Discussion

OKCs are developmental cysts arising from dental lamina or primordial tissues like the enamel organ [9]. Most (60%) of OKCs originate from dental lamina rests or oral epithelial basal cells, and the remaining 60% from the reduced enamel epithelium of the dental follicle.

OKCs have been reported to have a wide age distribution (8-82 years) with a mean age of occurrence in the second and third decades and a slight male predilection [8]. But in our case, the patient was a female in her fourth decade of life. OKCs may occur in any part of the upper and lower jaw, with the majority of cases occurring in the posterior mandible. However, in this case, the lesion was seen in the anterior lingual region of the mandible between the roots of teeth # 31 and 32.

Rarely, OKCs occur in the gingiva. Such lesions have been delegated as peripheral OKCs [12]. They are often misdiagnosed as lateral periodontal cysts, lateral radicular cysts, or gingival cysts of the adult. Even though the swelling in the present case mimicked a lateral periodontal cyst, it was clearly diagnosed as OKC on histopathological examination. Also, it was differentiated from peripheral OKC by the bony involvement observed in IOPA and CBCT images.

Literature reports very few cases of OKC mimicking a lateral periodontal cyst. Hiremath et al. in 2011 reported a case of an asymptomatic swelling of the gingiva between mandibular premolars and concluded that all pathologic specimens should be evaluated histopathologically for a definitive diagnosis due to the aggressive behavior and high recurrence rate of OKCs [12].

Bojan et al. in 2016 reported a case of OKC located between the roots of lower right premolars, which mimicked a lateral periodontal cyst, and concluded that the patient became asymptomatic one month postoperatively [13].

Thapa et al. reported a nodular mass in relation to the mandibular premolar area, which was provisionally diagnosed as a lateral periodontal cyst based on clinical and radiographic findings, which was later confirmed as OKC on histopathological examination [14].

Most cases of OKCs reported in the literature presented as swellings in the mandibular posterior region, with very few cases in the anterior region. To the best of our knowledge, this is the first case of OKC that presented as a swelling in the lingual aspect of mandibular anterior teeth.

Lateral periodontal cyst can be distinctly differentiated from OKC on histopathological examination. LPC is normally lined by thin, non-keratinized epithelium of one to five-layer thickness [8]. However, OKCs are generally lined by orthokeratinized or parakeratinized epithelium that is highly characteristic and has six to 10-layer ridges [8].

In the present case, parakeratinized epithelium was observed histologically. OKC is the most aggressive of all odontogenic cysts and shows the highest chance of recurrence. The recurrence rate varies between 2.5% and 62.5% [15]. Compared to classical OKCs, peripheral OKCs are clinically aggressive and have a higher chance of recurrence [16].

Parakeratotic type of OKCs have local aggressive behavior and a high recurrence rate. The histological picture of the parakeratotic type shows parakeratinized, stratified, squamous, corrugated epithelium and palisading basal cells as seen in the present case. This emphasizes the importance of regular and long-term follow-up of the patient. The patient is being regularly followed up, and complete bone regeneration is noted in the IOPA radiograph 36 months after surgery (Figure 4D).

Most keratocysts are asymptomatic. The most aggressive forms show a late buccal and lingual cortical bone expansion, as it has a tendency to invade the marrow. Lingual cortical plate expansion and perforation are not infrequent sequela. In later stages, inferior alveolar nerve involvement can also occur [3].

Diagnosis requires a surgical biopsy. The majority of clinicians favor “conservative” therapy, while others recommend more “aggressive” treatment options. Meiselman considered “enucleation, curettage, and marsupialization” as a conservative form of therapy [17]. Aggressive treatment connotes the “neoplastic nature” of OKC and comprises peripheral ostectomy, chemical curettage with Carnoy’s solution, or en bloc/segmental resection [18]. Many authors now advocate a more conservative approach and regular follow-up in treating the single non-syndromic OKC.

To fill the bony defect, in the present case, A-PRF was used. When compared to other platelet concentrates, A-PRF demonstrates prolonged growth factor release and has a higher concentration of platelets compared to leukocyte and platelet-rich fibrin (L-PRF). High elasticity and flexibility can be found in the A-PRF membrane. A-PRF was prepared by adhering to the 14-minute centrifugation time and 1500 rpm spinning rate technique [19]. The increased platelet concentration in A-PRF can potentially enhance the healing process [20].

In the present case, the cyst was situated between the central and lateral incisors, mimicking a lateral periodontal cyst. Histopathological examination of the cyst is mandatory for accurate diagnosis and treatment. OKC should always be considered in the differential diagnosis of swellings appearing lateral to a root because of its potentially aggressive behavior and high recurrence rate.

The follow-up recommendation for OKCs is once a year for a minimum of five years. Postoperative follow-up, along with radiographs, is essential annually for at least five years. Radical operations, like continuity resection, are not always warranted, as conservative management with marsupialization seems to work and preserves function with the least morbidity.

## Conclusions

Maxillofacial bone lesions can resemble each other clinically and radiographically. OKCs are often misinterpreted as lateral periodontal cysts. In the present case, the clinical presentation of the lesion, the radiographic appearance, and the typical histopathological findings helped in the final diagnosis. A correct diagnosis will help in the proper and timely management of the lesion, thereby preventing or minimizing recurrence. Hence, histopathological examination of all bone lesions should be performed for a definitive diagnosis.
